# COvid-19 and high-dose VITamin D supplementation TRIAL in high-risk older patients (COVIT-TRIAL): study protocol for a randomized controlled trial

**DOI:** 10.1186/s13063-020-04928-5

**Published:** 2020-12-28

**Authors:** Cédric Annweiler, Mélinda Beaudenon, Jennifer Gautier, Romain Simon, Vincent Dubée, Justine Gonsard, Elsa Parot-Schinkel, on behalf of the COVIT-TRIAL study group, Amal Aidoud, Guillaume Albaret, Cédric Annweiler, Alexandra Audemard-Verger, Marine Asfar, Jean Barré, Florian Berteau, Gaëlle Bertoletti, Jean-Baptiste Beuscart, Adrien Bigot, Sophie Boucher, Elisabeth Botelho-Nevers, Isabelle Bourdel-Marchasson, Anne Sophie Boureau, Antoine Brangier, Céline Brouessard, Marie Laure Bureau, Noëlle Cardinaud, Michel Carles, Karine Castro-Lionard, Thomas Celarier, Guillaume Chapelet, David Chirio, Emilie Clabé, Philippe Codron, Johan Courjon, Éric Cua, Marie Danet-Lamasou, Alexiane Decorbez, Marine De La Chapelle, Elisa Demonchy, Edouard Desvaux, Monique D’Hautefeuille, Vincent Dubée, Guillaume Duval, Bertrand Fougère, Paul Gassie, Nicolas Giroult, Olivier Guérin, Régis Hankard, Marjorie Houvet, Stéphanie Jobard, Carole Lacout, Aurélie Lafargue, Cécile Laubarie-Mouret, Maxime Le Floch, Sylvain Le Gentil, Sébastien Lléonart, Jocelyne Loison, Rafaël Mahieu, François Maillot, Laure Martinez, Marie Mathieu, Anthony Mauclere, Pierre Ménager, Emeline Michel, Thai Binh Nguyen, Romain Ordonez, Marie Otekpo, Virginie Pichon, Fanny Poitau, Gary Pommier, Valérie Rabier, Karine Risso, Hélène Rivière, Agnès Rouaud, Claire Roubaud-Baudron, Guillaume Sacco, Frédéric Scholastique, Etienne Seronie-Doutriaux, Achille Tchalla, Wojciech Trzepizur, Yves-Marie Vandamme

**Affiliations:** 1 Department of Geriatric Medicine and Memory Clinic, Research Center on Autonomy and Longevity, Angers University Hospital, F-49933 Angers, France; 2 UPRES EA 4638, University of Angers, Angers, France; 3 Robarts Research Institute, Department of Medical Biophysics, Schulich School of Medicine and Dentistry, The University of Western Ontario, London, ON Canada; 4 CRCINA, Inserm, Université de Nantes, Université d’Angers, 44200 Angers, Nantes France; 5 Service des Maladies Infectieuses et Tropicales, CHU, Angers, France; 6 Delegation for clinical research and innovation, Angers University Hospital, Angers, France; 7 Biostatistics and methodology department, Angers University Hospital, Angers, France

**Keywords:** COVID-19, SARS-CoV-2, Vitamin D, Anti-inflammatory, Antiviral, Randomized controlled trial, Older adults, Mortality, Prognosis

## Abstract

**Background:**

With the lack of effective therapy, chemoprevention, and vaccination against SARS-CoV-2, focusing on the immediate repurposing of existing drugs gives hope of curbing the COVID-19 pandemic. A recent unbiased genomics-guided tracing of the SARS-CoV-2 targets in human cells identified vitamin D among the three top-scoring molecules manifesting potential infection mitigation patterns. Growing pre-clinical and epidemiological observational data support this assumption. We hypothesized that vitamin D supplementation may improve the prognosis of COVID-19. The aim of this trial is to compare the effect of a single oral high dose of cholecalciferol versus a single oral standard dose on all-cause 14-day mortality rate in COVID-19 older adults at higher risk of worsening.

**Methods:**

The COVIT-TRIAL study is an open-label, multicenter, randomized controlled superiority trial. Patients aged ≥ 65 years with COVID-19 (diagnosed within the preceding 3 days with RT-PCR and/or chest CT scan) and at least one worsening risk factor at the time of inclusion (i.e., age ≥ 75 years, or SpO2 ≤ 94% in room air, or PaO2/FiO2 ≤ 300 mmHg), having no contraindications to vitamin D supplementation, and having received no vitamin D supplementation > 800 IU/day during the preceding month are recruited. Participants are randomized either to high-dose cholecalciferol (two 200,000 IU drinking vials at once on the day of inclusion) or to standard-dose cholecalciferol (one 50,000 IU drinking vial on the day of inclusion). Two hundred sixty participants are recruited and followed up for 28 days. The primary outcome measure is all-cause mortality within 14 days of inclusion. Secondary outcomes are the score changes on the World Health Organization Ordinal Scale for Clinical Improvement (OSCI) scale for COVID-19, and the between-group comparison of safety. These outcomes are assessed at baseline, day 14, and day 28, together with the serum concentrations of 25(OH)D, creatinine, calcium, and albumin at baseline and day 7.

**Discussion:**

COVIT-TRIAL is to our knowledge the first randomized controlled trial testing the effect of vitamin D supplementation on the prognosis of COVID-19 in high-risk older patients. High-dose vitamin D supplementation may be an effective, well-tolerated, and easily and immediately accessible treatment for COVID-19, the incidence of which increases dramatically and for which there are currently no scientifically validated treatments.

**Trial registration:**

ClinicalTrials.govNCT04344041. Registered on 14 April 2020

**Trial status:**

Recruiting. Recruitment is expected to be completed in April 2021.

## Background

Since December 2019, the COVID-19 caused by SARS-CoV-2 is spreading worldwide, affecting millions of people and leaving hundreds of thousands dead. With the lack of effective therapy, chemoprevention, and vaccination [1], focusing on the immediate repurposing of existing drugs gives hope of curbing the pandemic. Importantly, a recent unbiased genomics-guided tracing of the SARS-CoV-2 targets in human cells identified vitamin D among the three top-scoring molecules manifesting potential infection mitigation patterns through their effects on gene expression [2].

Vitamin D is a secosteroid hormone [3, 4]. By binding to the vitamin D response elements (VDRE) located in the promoter region of various genes, the expression of which is either activated or repressed, vitamin D may prevent COVID-19 adverse outcomes by regulating (i) the renin-angiotensin system (RAS), (ii) the innate and adaptive cellular immunity, (iii) the physical barriers, and (iv) the host frailty and comorbidities [5]. First, vitamin D reduces pulmonary permeability in animal models of acute respiratory distress syndrome (ARDS) by modulating the activity of RAS and the expression of the angiotensin-2 converting enzyme (ACE2) [6, 7]. This action is crucial since SARS-CoV-2 appears to use ACE2 as a receptor to infect host cells [8] and downregulates ACE2 expression [9]. ACE2 is expressed in many organs, including the endothelium and the pulmonary alveolar epithelial cells, where it has protective effects against inflammation. During COVID-19, downregulation of ACE2 results in an inflammatory chain reaction, the cytokine storm, complicated by ARDS [10, 11]. On the contrary, a study in rats with chemically induced ARDS showed that the administration of vitamin D increased the levels of mRNA and proteins ACE2 [12]. Rats supplemented with vitamin D had milder ARDS symptoms and moderate lung damage compared to controls. Second, many studies have described the antiviral effects of vitamin D, which works either by induction of antimicrobial peptides with direct antiviral activity against enveloped and non-enveloped viruses or by immunomodulatory and anti-inflammatory effects [13, 14]. These are potentially important during COVID-19 to limit the cytokine storm. Vitamin D can prevent ARDS [15] by reducing the production of pro-inflammatory Th1 cytokines, such as TNFα and interferon γ [13]. It also increases the expression of anti-inflammatory cytokines by macrophages [13]. Third, vitamin D stabilizes physical barriers [5]. These barriers are made up of closely linked cells to prevent outside agents (such as viruses) from reaching tissues susceptible to infection. Although viruses alter the integrity of the cell junction, vitamin D contributes to the maintenance of functional tight junctions via E-cadherin [5]. Fourth, the literature over the past decade on the non-skeletal effects of vitamin D has repeatedly reported that hypovitaminosis D is accompanied by various comorbidities including diabetes mellitus, hypertension, chronic cardiovascular and respiratory diseases, and cancers [3], all conditions that are associated with an increased risk of COVID-19 worsening and death.

Vitamin D is naturally synthesized by the skin during summer exposure to solar ultraviolet B rays. Hypovitaminosis D in humans is therefore more frequent in winter from October to March in the northern latitudes (above 20°) [3], which corresponds precisely to the latitudes where the highest fatality rates of COVID-19 were observed in the first winter months of 2020 [1]. In the past, coronaviruses and influenza viruses have shown a very strong seasonality, with preferential winter appearances in the northern hemisphere [16]. It should also be noted that the mortality of COVID-19 disproportionately affects BAME individuals (Blacks, Asians, and Ethnic Minorities) [17], whose higher cutaneous levels of melanin reduce the potential for cutaneous vitamin D synthesis.

The first reports indicated that cases with COVID-19 had, on average, 25(OH)D concentrations more than twice as low as controls without COVID-19 (respectively, 11.1 ng/mL versus 24.6 ng/mL, *P* = 0.004) [18]. Similarly, significant inverse correlations were found in 20 European countries between the mean serum 25(OH)D concentrations and the number of COVID-19 cases, as well as with mortality [19]. The severity of hypovitaminosis D appears to relate to the prognosis of COVID-19 since COVID-19 cases with hypovitaminosis D were more prone to experience severe COVID-19 (relative risk 1.59 with *P* = 0.02 if vitamin D insufficiency < 30 ng/mL) [20]. Finally, hypovitaminosis D was found to be associated with greater COVID-19 mortality risk (IRR = 1.56 with *P* < 0.001 if vitamin D deficiency; *P* = 0.404 after adjustment) [21]. These results support that enhancing serum 25(OH)D concentration may improve the prognosis of COVID-19, as strengthened by a pilot controlled trial reporting that the administration of calcifediol versus no calcifediol reduced the need for ICU treatment in 76 hospitalized participants with COVID-19 also receiving the best available therapy (mean age, 53 years; 40.8% women) [22]. Similarly, two quasi-experimental studies reported higher 14-day survival rates in frail older adults with COVID-19 receiving regular [23] and preferentially recent [24] vitamin D supplementation. Following these preliminary findings, larger interventional studies dedicated to COVID-19 are warranted for investigating the role of vitamin D supplementation on COVID-19 outcomes. Interestingly, previous meta-analyses revealed that high-dose prophylactic vitamin D supplementation was able to reduce the risk of respiratory tract infections [25, 26]. The optimal dose varied between 1000 and 4000 IU/day, suggesting the benefits of high-dose vitamin D supplementation, as did a previous study in an intensive care unit that reported a 17% mortality rate reduction in patients who received high-dose vitamin D compared to a placebo [27]. It is considered safe to take oral vitamin D supplements up to 10,000 IU/day for short periods [28], especially in older adults, a population predominantly affected by hypovitaminosis D and who should receive at least 1500 IU/day of vitamin D to get a desirable vitamin D status [3, 4].

We hypothesized that high-dose vitamin D supplementation could improve the prognosis of COVID-19.

## Methods

### Objectives

The primary objective is to compare the effect of a single oral high dose of cholecalciferol versus a single oral standard dose of cholecalciferol on 14-day all-cause mortality rate in older adults infected with SARS-CoV-2 at higher risk of worsening.

The secondary objectives of the study are as follows:

- To compare the effect of high-dose versus standard-dose cholecalciferol on 28-day all-cause mortality rate
- To compare the effect of high-dose versus standard-dose cholecalciferol on the clinical course of the disease (i.e., change of World Health Organization’s [WHO] Ordinal Scale for Clinical Improvement [OSCI] scale for COVID-19) within 14 and 28 days of inclusion [29]
- To compare the safety of high-dose versus standard-dose cholecalciferol
- To compare the effect of high-dose versus standard-dose cholecalciferol on all-cause mortality rate and OSCI scale changes within 14 and 28 days of inclusion in the subgroup with vitamin D deficiency at baseline (i.e., serum 25(OH)D < 25 nmol/L)
- To determine the impact of serum 25(OH)D concentration at day 7 (i.e., serum 25(OH)D concentration < 75 nmol/L versus ≥ 75 nmol/L) on mortality rate and OSCI scale changes within 14 and 28 days of inclusion
- To determine, in the subgroup with vitamin D deficiency at baseline (i.e., serum 25(OH)D < 25 nmol/L), the impact of the serum 25(OH)D concentration reached at day 7 (i.e., serum 25(OH)D concentration < 75 nmol/L versus ≥ 75 nmol/L) on mortality rate and OSCI scale changes within 14 and 28 days of inclusion
- To model survival and clinical course (OSCI scale changes) within 14 and 28 days of inclusion according to the change of serum 25(OH)D concentration between day 0 and day 7
- To compare the mortality rates observed within 14 days of inclusion in each arm of the study with the mortality rates reported in the French hospital geriatric units receiving COVID-19 patients

### Design

**Fig. 1:**
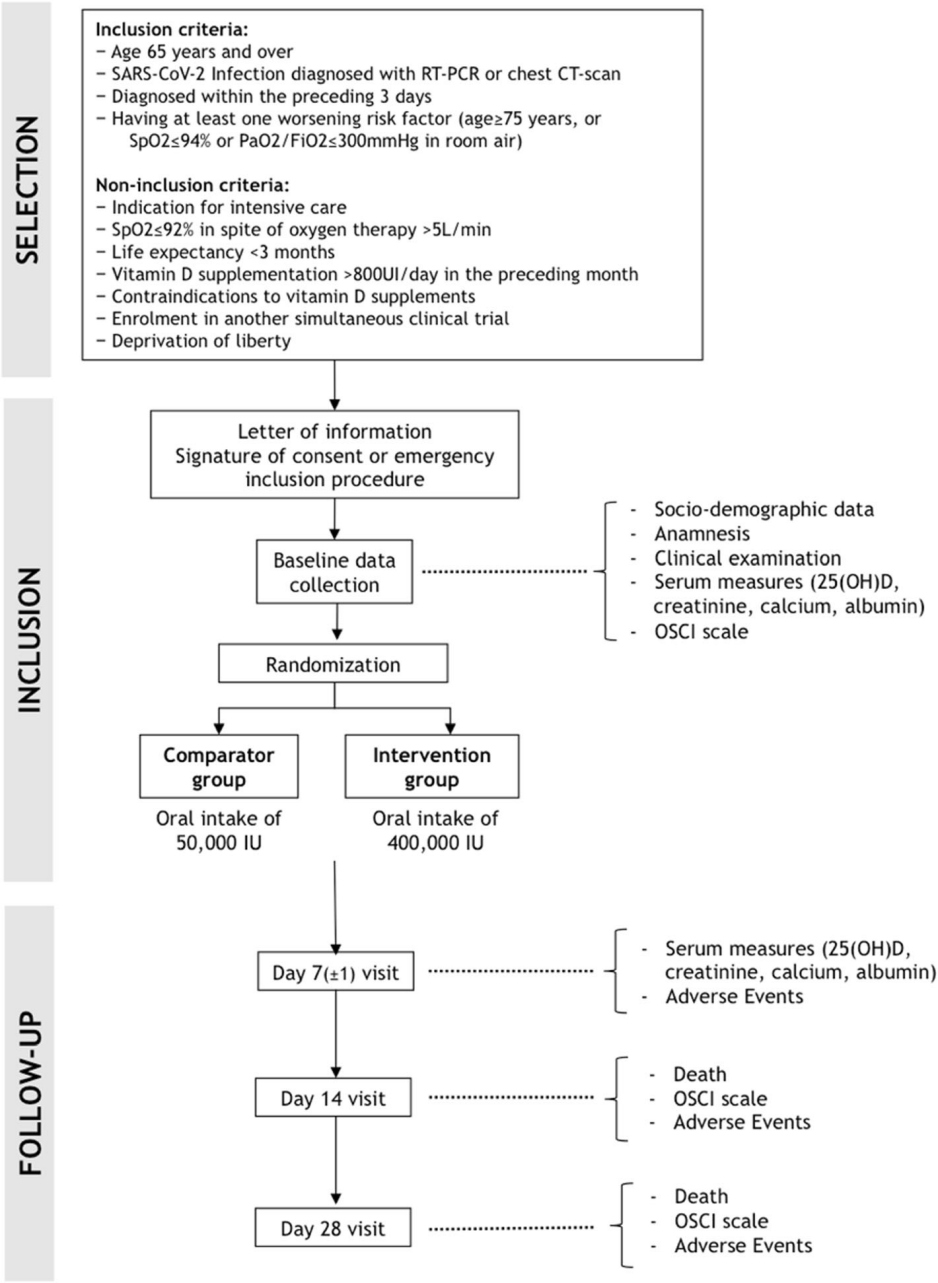
Trial flow chart

This is an open-label, multicenter, randomized, controlled, parallel group, intent-to-treat, superiority clinical trial. Figure 1 illustrates the trial design, and Table 1 summarizes the timing of the trial. All participants receive the intervention in a single oral intake on the day of inclusion. Treatment allocation is carried out according to a 1:1 ratio by means of dynamic randomization (randomization by minimization) taking into account 6 criteria: worsening risk factor (age ≥ 75 years, or oxygen dependence characterized by a peripheral capillary oxygen saturation [SpO2] ≤ 94% in room air, or ratio of partial oxygen pressure [PaO2] to oxygen fraction in the inspired air [FiO2] ≤ 300 mmHg), criterion used for COVID-19 diagnosis (RT-PCR or chest CT scan), hospitalization (yes/no), concomitant use of anti-infective drugs such as hydroxychloroquine or azithromycin among others, concomitant use of corticosteroids, and recruiting center. This dynamic randomization is established by the Department of Biostatistics and Methodology of the University Hospital of Angers, France. Randomization is performed using a web-based system (Ennov Clinical®).

**Table 1:** Calendar summary

	Study period			
	Enrolment and allocation	Post-allocation		Close-out
Timepoint	Day 0	Day 7 (± 1 day)	Day 14	Day 28
**Enrolment**				
Eligibility screen	X			
Inclusion (written consent or emergency inclusion procedure)	X			
Allocation	X			
**Interventions**				
High-dose vitamin D supplementation	X			
Standard-dose vitamin D supplementation	X			
**Assessments**				
Socio-demographic, clinical, treatment data	X		X	X
Blood test for serum 25(OH)D, creatinine, calcium, and albumin	X	X		
OSCI scale	X		X	X
AE/SAE		X	X	X
All-cause death		X	X	X

Treatments were transmitted to the central pharmacy of Angers University Hospital, which is in charge of shipping the treatments to each recruiting center taking into account the pace of inclusion within each center. Study medication packs are identified by means of random numbers. The Ennov Clinical® software assigned to the patient a study medication pack (random number) corresponding to the result of the dynamic randomization.

#### “Intervention” group

All participants receive the high-dose cholecalciferol (vitamin D_3_) supplement in a single oral intake on the day of inclusion (Mylan; 75008 Paris, France). Cholecalciferol is packaged in a drinking vial of 2 mL containing 200,000 IU. Thus, participants in the Intervention group take two vials at once, to reach the studied total dose of 400,000 IU. Vitamin D is ideally taken during food intakes because vitamin D is lipophilic and therefore better absorbed with fat. Such high dose is expected to quickly raise the serum 25(OH)D concentration above 75 nmol/L [30], which is not expected with lower doses [23]. This is all the more important in that, based on the knowledge of vitamin D non-skeletal effects, it appears that raising serum 25(OH)D concentrations above 75 nmol/L is necessary to obtain antiviral effect [4]. Finally, it has to be quoted that the chosen dose of 400,000 IU does not reach the dose of 500,000 IU that has been previously associated with side effects [31]. In addition, participants in the Intervention group will not take other vitamin D supplements for 45 days and will therefore receive the equivalent of a daily dose of 8900 IU/day over the period, less than the toxic dose of 10,000 IU/day [28].

#### “Comparator” group

All participants receive the standard-dose cholecalciferol (vitamin D_3_) supplement in a single oral intake on the day of inclusion (Mylan; 75008 Paris, France). The treatment is packaged in a drinking vial of 2 mL containing 50,000 IU. Thus, participants in the Comparator group take one vial, to reach the studied total dose of 50,000 IU, in accordance with the maintenance treatment recommended in 2020 by the French Osteoporosis Research and Information Group (GRIO) [32]. The appearance and the number of drinking vials varying between the two arms, the study is open-labeled and both the investigators and the participants know which supplementation dose is taken.

We have chosen not to use a placebo in the study because it would not be legitimate in older adults since (i) 80 to 100% of non-supplemented older adults have hypovitaminosis D [4], (ii) hypovitaminosis D is accompanied by a high number of potentially serious acute and chronic diseases that are associated with higher mortality in COVID-19 patients [3, 4], and (iii) an international scientific society recommends vitamin D supplementation in primary prevention of COVID-19 in older adults [33]. Based on previous literature [27], we have also considered unlikely that the placebo effect of taking high-dose vitamin D is able to prevent death from COVID-19, even if our open-label study will not eliminate this plausibility.

Enrolled participants are also provided with oral and written information that they should not be prescribed medication containing vitamin D outside of the trial for the duration of the study (Comparator group) and up to 45 days for the Intervention group.

### Planned eligibility criteria

#### Inclusion criteria

People are eligible to participate if they are 65 years and over, if they have a SARS-CoV-2 infection diagnosed within the preceding 3 days by RT-PCR and/or chest CT scan, and if they have at least one worsening risk factor (age ≥ 75 years, or SpO2 ≤ 94% in room air, or PaO2/FiO2 ≤ 300 mmHg). Participants must be covered by or having the rights to medical care insurance. Participants must also have given and signed an informed consent form to participate in the trial (or informed consent form obtained from the trusted person, or emergency inclusion procedure in the context of lockdown, as appropriate).

#### Non-inclusion criteria

The following non-inclusion criteria are considered: organ failure requiring admission to intensive care, SpO2 ≤ 92% despite oxygen therapy > 5 L/min, life expectancy < 3 months, any reason preventing follow-up at 28 days, treatment with vitamin D supplementation during the preceding month (with the exception of supplements providing 800 IU or less vitamin D per day), contraindications to the use of vitamin D supplements (active granulomatosis [sarcoidosis, tuberculosis, lymphoma], history of calcium lithiasis, known hypervitaminosis D or hypercalcemia, known intolerance to vitamin D), enrolment in another simultaneous clinical trial, and deprivation of liberty by an administrative or judicial decision.

### Recruitment/consent procedures

Participants are identified from patients with COVID-19 who were diagnosed in each hospital and/or nursing home recruiting center. The 10 recruiting centers are all located in France and include Angers University Hospital, Bordeaux University Hospital, Le Mans Hospital, Lille University Hospital, Limoges University Hospital, Nantes University Hospital, Nice University Hospital, Saumur University Hospital, Saint-Etienne University Hospital, and Tours University Hospital. The participation of 4 additional French centers is under study (Château-Gontier Hospital, Lyon University Hospital, Public Assistance Hospital of Paris – Broca Hospital, Rennes University Hospital). Once a potential participant is identified and meets the eligibility criteria, the investigating physician provides the patient and relatives written and oral information on the study in an understandable language and obtains written consent to take part in the study in line with the trial standard operating procedures. When possible, fully informed consent is obtained from the patient. When a patient is unable to give fully informed consent, agreement to participate in the study is obtained from the trusted person, and the patient is not enrolled if s/he refuses or shows significant distress. If it is impossible for the trusted person to sign the consent due to nationwide lockdown, an emergency inclusion procedure may be conducted and will be accompanied by a consent returned by post as soon as possible. In case of refusal to participate in the study, the investigating physician will record the cause of non-participation in the study and copy it in the “Registry of non-eligible and eligible patients.”

### Assessments

Study assessment measures will be applied at baseline prior to randomization, at day 7 (± 1 day), day 14, and day 28. All measures in the study are usually carried out systematically in every patient admitted to the recruiting centers. Experience has shown that this operational mode is feasible and appreciated by patients and their relatives and has never caused a breakdown in care so far [34]. For this reason, the number of measures is not expected to exacerbate the loss to follow-up. Phone calls are planned to keep in touch with the participants. Trial completion is defined as completion of 28 days or discontinuation of follow-up for any cause.

#### Primary outcome measure

The primary outcome measure is all-cause mortality within 14 days of inclusion. Such follow-up time has the merit of including the mortality risk linked to the cytokine storm that usually occurs between the 7th and 10th day after infection [1], the clinical expression of which is fatal ARDS. Moreover, this objective outcome measure does not give rise to monitoring or measurement bias despite the open-label design.

#### Secondary outcome measures

The secondary outcome measures are (i) all-cause mortality within 28 days of inclusion and (ii) the changes of WHO’s OSCI scale for COVID-19 between baseline and day 14 and between baseline and day 28 [29].

#### Blood sample collection procedures

All participants are tested for 25(OH)D concentration at baseline and day 7 (± 1 day). Blood tests are done by a clinical research nurse. Serum 25(OH)D concentration is measured with radioimmunoassay (RIA) kits in each recruiting center. RIA kits recognize both vitamins D_2_ and D_3_. With this method, there is no interference of lipids, which is often observed in other non-chromatographic assays of 25(OH)D. The intra- and interassay precisions are respectively 5.2% and 11.3% (range in normal adults aged 20–60 years, 75–310 nmol/L). Creatinine, calcium, and albumin (to estimate corrected calcemia) are also measured at baseline and day 7 (± 1 day) with standard laboratory methods.

#### Safety parameters

The safety assessment parameters are:

- Clinical: asthenia, vertigo, headache, anorexia, nausea and vomiting, polyuria-polydipsia, lumbar or right hypochondrium pain in case of biliary or renal colic revealing calcic lithiasis (objectified by ultrasound in the case of clinical suspicion)
- Biological: serum calcium, albumin (to estimate corrected calcemia), and creatinine concentrations at baseline and day 7 (± 1 day). The finding of hypercalcemia gives rise to appropriate care and triggers further complementary non-specific examinations to find the cause of hypercalcemia for which vitamin D supplementation could not be held responsible at first [3].

### Statistics

#### Sample size calculation

The trial aims to recruit 260 patients divided into 2 groups of 130 patients. Based on previous literature, the expected death rate in the standard-dose vitamin D group is estimated at 20% in this population with worsening risk factors [35]. A total of 125 participants per group should be involved to demonstrate, under a bilateral hypothesis, a between-group absolute difference in all-cause mortality risk of 12% with an alpha risk of 5% and a power of 80%. Such target is consistent with one previous study in an intensive care unit reporting a 17% mortality rate reduction in patients who received high-dose vitamin D compared to a placebo [27]. Taking into account the loss to follow-up (estimated at 5%), it is necessary to include a total of 260 subjects (130 per group).

#### Analyses

The effect of high-dose vitamin D supplementation compared to standard-dose vitamin D supplementation will be determined using evaluation criteria, which are the mortality rates and the changes in OSCI score within 14 and 28 days of inclusion, using respectively the chi-square test or exact Fischer test, and independent samples *t* test or Mann-Whitney *U* test, as appropriate. An interim analysis of 100 participants is planned. It will focus on the primary outcome measure (i.e., all-cause mortality within 14 days of inclusion) with a *P* value of 0.001 used as the significance threshold for stopping the study. The final analysis of the primary outcome measure will use the *P* value of 0.0498 as the significance threshold (Peto-Haybittle method).

The proportions of participants with at least one serious adverse event within 28 days of inclusion will be compared using the chi-square test (or exact Fisher test if necessary). Survival curves will be plotted from baseline to day 28 using the Kaplan-Meier method, and a Cox proportional hazards model will be used to compare clinical outcomes between groups. The log-linearity assumptions will be checked for each quantitative variable introduced in the Cox models, and proportional hazards assumption will be checked for each variable to confirm adequacy for the Cox models.

Any exploratory analyses here will use multivariate logistic and proportional hazards regression. In addition, the stratification criterion for randomization will be also taken into account in the multivariate models. Excessive subgroup analyses can give rise to misleading results, and therefore, all subgroup investigations will be interpreted cautiously. In particular, subgroup analysis will be conducted depending on the severity of hypovitaminosis D at baseline and at day 7.

### Ethical considerations

The protocol received approval from the French “Sud-Est V Ethics Committee” (20.04.03.65603, Grenoble, France; ref., 20-ANGE-01) and was also approved by the French National Agency for Medicines and Health Products Safety (ANSM). The trial is conducted in compliance with French law no. 2012-300 of 12 March 2012 relative to the researches involving the human person and following Good Clinical Practice (I.C.H. E6(R2)), the Public Health Code of Ethics, the French National Commission for Information Technology and Freedom (1978), and the Helsinki Declaration (Ethical Principles for Medical Research involving Human Subjects, Tokyo 2004) and other requirements as appropriate. A verification of the consents and emergency procedures is carried out after each inclusion, followed by an auditing of the files at regular time intervals.

An Independent Data Monitoring Committee (IDMC), independent from the sponsor and competing interests, will monitor the progress of the trial including recruitment, serious adverse events, and side effects of treatment as well as the difference between the trial treatments on the primary outcome measures. The IDMC will produce a report to the Trial Steering Committee (TSC) after every meeting and can recommend premature closure of the trial following clear evidence of benefit or harm in accordance with the IDMC charter.

Individual expected benefits are:

- All participants receive vitamin D supplements, in line with IAGG guidelines [33], while 80 to 100% of those meeting eligibility criteria (i.e., receiving no regular significant vitamin D supplementation) exhibit hypovitaminosis D before the study [3, 4].
- The expected benefits are potentially important because, in the hypothesis of an effect of vitamin D supplementation in COVID-19, participation in this research may prevent mortality rate and improve COVID-19 outcomes, in addition to the prevention of health issues that usually accompany hypovitaminosis D.

The expected collective benefits for this research are an improvement of the knowledge of the non-skeletal effects of vitamin D. High-dose vitamin D supplementation may represent an effective, accessible, and well-tolerated treatment for COVID-19, the incidence of which increases dramatically and for which there are currently no scientifically validated treatments. The results of the study should be available before the end of the pandemic and should therefore benefit all older adults infected with SARS-CoV-2 in France and abroad. However, since the COVIT-TRIAL study is designed to reveal a relatively high decrease in mortality rate of 12%, it is possible that the study is underpowered to find more subtle effects of vitamin D on COVID-19 outcomes, and further larger randomized clinical trials would be deemed necessary in the event of non-significant results here.

## Discussion

No specific treatments for COVID-19 exist right now. With confirmed COVID-19 cases worldwide reaching millions and continuing to grow, there is an unprecedented research effort to develop treatments and vaccines to slow the pandemic and lessen the disease’s damage. As of 8 May 2020, more than 1000 clinical trials seek to assess dozens of potential treatments [https://covid-trials.org], including 340 in China, 185 in the USA, and 69 in France.

One of the main objectives for coping with the COVID-19 pandemic is to identify an active treatment on SARS-CoV-2 that will control the infection and, if possible, reduce the progression to serious forms that are potentially fatal during COVID-19. Two main therapeutic strategies are explored: combating viral replication of SARS-CoV-2 (with hydroxychloroquine for example) or limiting the reactive cytokine inflammatory storm (with corticosteroids for example) [1]. No treatment is currently validated in the therapeutic management of COVID-19, even if the administration of systemic corticosteroids has shown some interest on mortality risk in critically ill patients with COVID-19 [36]. The difficulty is to associate scientific methodological rigor with the public health emergency in controlling the pandemic. Interestingly, the COVIT-TRIAL study brings together all these criteria, by proposing a rigorous protocol meeting the best standards of clinical research to provide a response with the highest level of evidence about the effect on COVID-19 outcomes of high-dose vitamin D supplements, a molecule with pleiotropic properties theoretically capable of limiting both viral replication and cytokine storm [5], together with preventing several comorbidities responsible for mortality in individuals infected with SARS-CoV-2 [3].

Growing pre-clinical and clinical evidence support vitamin D as a biological determinant of COVID-19 outcomes. In the absence of preventive or curative treatment, several scientific societies have not waited for interventional data to recommend supplementing older adults with vitamin D to prevent the onset of COVID-19 [29]. According to the clinicaltrials.gov database, six other clinical trials are in preparation to test the effect of vitamin D supplements during or in the prevention of COVID-19 but, as of 8 May 2020, none has started to recruit participants yet. Thus, future publications on the primary and secondary results of the COVIT-TRIAL study will make a substantial contribution to this important orientation of research, and the results will provide clear evidence on the effect of high-dose vitamin D supplementation in significantly improving the clinical presentation of COVID-19 and its prognosis. However, if the results were found to be not significant here, further clinical trials with larger sample size and various supplementation regimens, possibly adjuvanting other active treatments, would be deemed necessary to determine whether vitamin D supplementation allows for more subtle reduction in mortality during COVID-19.

### Trial status

COVIT-TRIAL version 5 on 17 July 2020. Recruitment began on 15 April 2020 and is expected to be completed by April 2021.

## Data Availability

The principal investigator, Cedric Annweiler, MD, PhD, will have access to the final trial dataset.
